# Rosemary and neem: an insight into their combined anti-dandruff and anti-hair loss efficacy

**DOI:** 10.1038/s41598-024-57838-w

**Published:** 2024-04-02

**Authors:** Mona M. Hashem, Dalia Attia, Yomna A. Hashem, Moataz S. Hendy, Safa AbdelBasset, Farah Adel, Maha M. Salama

**Affiliations:** 1 Department of Pharmacognosy, Faculty of Pharmacy, Cairo University, Kasr El-Aini Street, Cairo, 11562 Egypt; 2 Department of Pharmaceutics and Pharmaceutical Technology, Faculty of Pharmacy, The British University in Egypt, Suez Desert Road, El Sherouk City, Cairo, 11837 Egypt; 3 Department of Microbiology, Faculty of Pharmacy, The British University in Egypt, Suez Desert Road, El Sherouk City, Cairo, 11837 Egypt; 4 Department of Pharmaceutical Chemistry, The British University in Egypt, Suez Desert Road, El Sherouk City, Cairo, 11837 Egypt; 5 Health Research Centre of Excellence, Drug Research and Development, The British University in Egypt, Suez Desert Road, El Sherouk City, Cairo, 11837 Egypt; 6 Department of Pharmacognosy, Faculty of Pharmacy, The British University in Egypt, Suez Desert Road, El Sherouk City, Cairo, 11837 Egypt

**Keywords:** Dandruff, Rosemary herb, Neem leaves, Herbal formula, Antimicrobial, Anti-inflammatory, Drug discovery, Plant sciences

## Abstract

Dandruff, a common scalp disorder characterized by flaking dead skin, is often treated with conventional topical products. However, limitations exist due to potential side effects and high costs. Therefore, searching for natural, cost-effective solutions for dandruff and hair loss is crucial**.** Rosemary herb and neem tree, both cultivated in Egypt, possess well-documented anti-inflammatory properties derived from their rich phenolic phytoconstituents**.** This study formulated a standardized combined extract of rosemary and neem (RN-E 2:1) into hair gel and leave-in tonic formats. This extract demonstrated superior efficacy against *Malassezia furfur* (a causative agent of dandruff) and *Trichophyton rubrum* (associated with scalp disorders) compared to the conventional antifungal agent, ketoconazole. The combined extract (RN-E 2:1) also exhibited potent anti-inflammatory activity. Additionally, the suppression of iNOS expression is considered concentration-dependent. Quality control verified formulation stability, and ex-vivo studies confirmed effective ingredient penetration into the epidermis, the primary site of fungal presence. Remarkably, both formulations outperformed the standard treatment, minoxidil in hair growth trials. These findings highlight the potential of natural extracts for scalp and hair health.

## Introduction

*Pityriasis capitis*, commonly known as dandruff, is a disease restricted to the scalp that has been around for centuries despite several treatment options. Half of adults (especially men) experience dandruff, characterized by white flakes, mild itching, and potentially progressing to inflamed scalp (seborrheic dermatitis) and even hair loss (alopecia)^1^. Individuals with dandruff experience diminished self-confidence and self-esteem due to the presence of visible scalp itching, flaking, and scaling. Notably, the severity of this chronic scalp condition exhibits seasonal fluctuations, often worsening during winter months. Furthermore, the economic burden of dandruff is substantial, with annual expenditures exceeding $300 million in the United States alone^2^.

Scalp belongs to the integumentary system that protects and covers the human head. It contains normal flora where about one billion microorganisms inhabit a square centimeter of the human skin. Microorganisms include bacteria, fungi, and viruses, developing a complex community known as the skin microbiome or normal flora^3^. These microorganisms, especially *Malassezia* yeast, lack the enzymatic system necessary for fatty acid synthesis. Consequently, they depend on exogenous sources to fulfill their fatty acid requirements. Malassezia yeasts secrete various lipases, enzymes capable of hydrolysis triglycerides present in sebum, a lipid-rich secretion of sebaceous glands.

Normal flora comprises beneficial microorganisms that colonize the superficial layer of the skin and the top parts of the hair follicles. The diverse microbial ecosystem of the scalp, known as the scalp microbiome, is subject to both extrinsic and intrinsic influences that affect its composition and function. Extrinsic factors, such as sun exposure, stress, dry skin, seborrheic dermatitis, unsuitable cosmetic use, and humidity, can all perturb the scalp microbiome. Intrinsic factors, including dietary imbalances, genetic susceptibility towards specific microbiota communities, immunosuppression, and hormonal fluctuations, also play a significant role in shaping the scalp microbiome. An insight into the complex interchange between these extrinsic and intrinsic factors and their impact on the scalp microbiome is crucial for developing effective strategies to maintain scalp health and prevent or manage dermatological disorders^4^. Therefore, any imbalance between these microorganisms can cause many undesired scalp disorders, especially dandruff and hair fall^5^. Microorganisms found on the scalp are *Propionibacterium acnes*, Dandruff affects almost half of the population, especially adults, it is more prominent in males than females. The condition is manifested by dead skin exfoliating *Staphylococcus epidermidis* and most commonly *Malassezia* species. These microorganisms secrete are considered opportunistic pathogens in case of body disorders^6^. These microorganisms especially *Malassezia* are incapable of synthesizing fatty acids, leading them to rely on external sources for this crucial nutrient**,** *Malassezia* compensate for this dependence by secreting lipases, enzymes capable of breaking down triglycerides present in sebum, a lipid-rich secretion of the sebaceous glands. This enzymatic activity can potentially contribute to the peroxidation of sebum triglycerides, resulting in the formation of arachidonic acid**.** Arachidonic acid promotes keratinocyte proliferation and differentiation to release pro-inflammatory cytokines (TNF-*α* and interleukin 1α, 6, 8) hair occlusion and rupture accompanied by erythema, pruritus, and scaling^7,8^. *Trichophyton rubrum*—fungi -can penetrate and invade the outer root sheath of the hair follicle causing *Tinea capitis,* which is characterized by itching, dry scaly areas, redness, and finally hair loss^9^.

Conventional treatments for dandruff and hair fall (topical/systemic) are extensively employed, these include keratolytic agents, anti-inflammatory, and antimicrobial agents^10,11^. However, cost considerations and potential adverse effects limit their use in some cases^4,12,13^. The need for novel and reliable methods for dandruff and hair fall management is crucial. Medicinal plants are gifts to human society to combat diseases with low side effects; plant extracts, and essential oils, are drugs rich in biologically active metabolites with potent antioxidant and anti-microbial activity in addition to their anti-inflammatory activity^14^. Furthermore, several plant extracts have been described for the management of dandruff and hair loss due to their antioxidant properties, either used alone or combined with other natural ingredients, others are added in a combinatorial formula to potentiate other conventional drugs or to decrease their side effects^15,16^.

Rosemary (*Rosmarinus officinalis* L. *Salvia Rosmarinus*) *F. Lamiaceae* is a fragrant evergreen herb native to the Mediterranean region and cultivated in Egypt. It is recognized for its valuable phytoconstituents; carnosic and rosmarinic acid, volatile oil, and phenolics along with their derivatives that have been implemented in many pharmaceutical and cosmetic products, an application which has been established and appreciated since Ancient Egyptian times^17^. These metabolites have also been evidenced to have antioxidant, anti-microbial, anti-inflammatory, neuroprotective, and anti-diabetic activity^18,19^*.* The neem tree, also known as *Azadirachta indica* (nimtree), belongs to the mahogany family (Meliaceae). This tree thrives in semi-tropical and tropical regions and is even cultivated in Egypt. Neem tree is considered an important source of distinctive compounds such as volatile, tannins and phenolic compounds, terpenoids, and steroids that play a role in developing medicines against several diseases/disorders and enhancing pharmaceutical products^20^.

Based on the reported activity of both plants, their availability, and their ease of access, we aim to formulate anti-dandruff/hair loss in different pharmaceutical products from a combined standardized ethanolic extract of Rosemary herb and Neem leaves extract (RN-E) in different dosage forms with multi-targeted approach: an antioxidant, antifungal agents, anti-inflammatory, with fewer side effects.

## Results

### Total Phenolic and flavonoid contents

The concentrations of total phenolics and flavonoids were ascertained by employing standard calibration curves for gallic acid and rutin, as illustrated in Supplementary Fig. S1 online. Rosemary and neem ethanolic extracts exhibited total phenolic content values of 96.7 ± 4 and 29.5 ± 7.8 µg gallic acid equivalent/mg, respectively. In contrast, rosemary and neem contained total flavonoid contents of 4.49 ± 0.32 and 3.49 ± 0.41 µg rutin equivalent/mg, respectively. These results revealed the richness of rosemary and neem with phenolic and flavonoid contents. Accordingly, a combined extract of rosemary: neem (RN-E 2:1) was prepared and tested for their total phenolic and flavonoid contents which give a value of 104.4 ± 8 µg GA eq/mg and 4.485 ± 0.08 µg rutin eq/mg total phenolic and total flavonoid contents, respectively. The observed findings suggest a potential synergy between the two extracts that potentiate each other, suggesting their possible anti-inflammatory and antioxidant power.

### UPLC-PDA standardization

**Figure 1:**
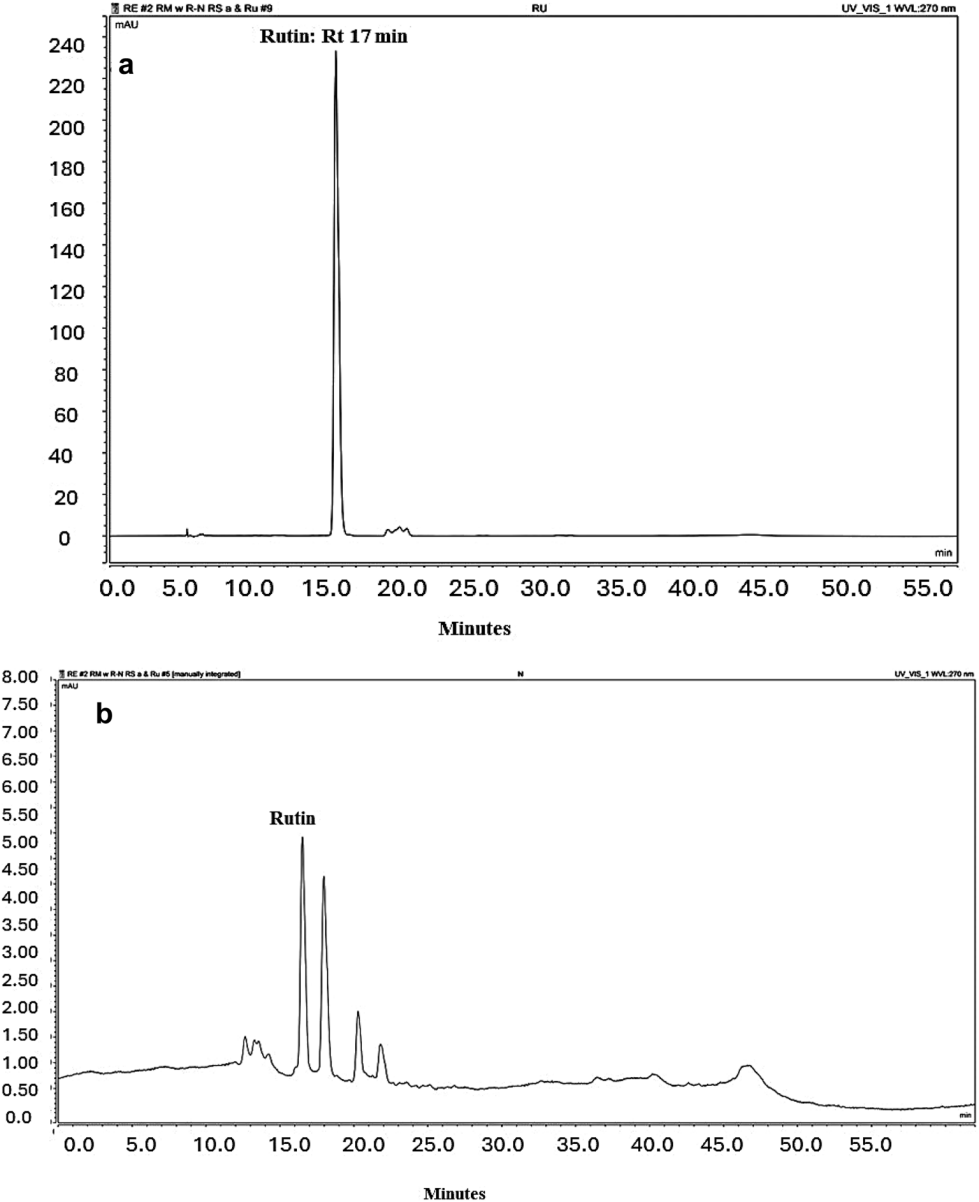
HPLC chromatograms; (**a**) Chromatogram of rutin standard (Rt:17 min); (**b**) fingerprint chromatogram of neem leaf ethanolic extract.

**Figure 2:**
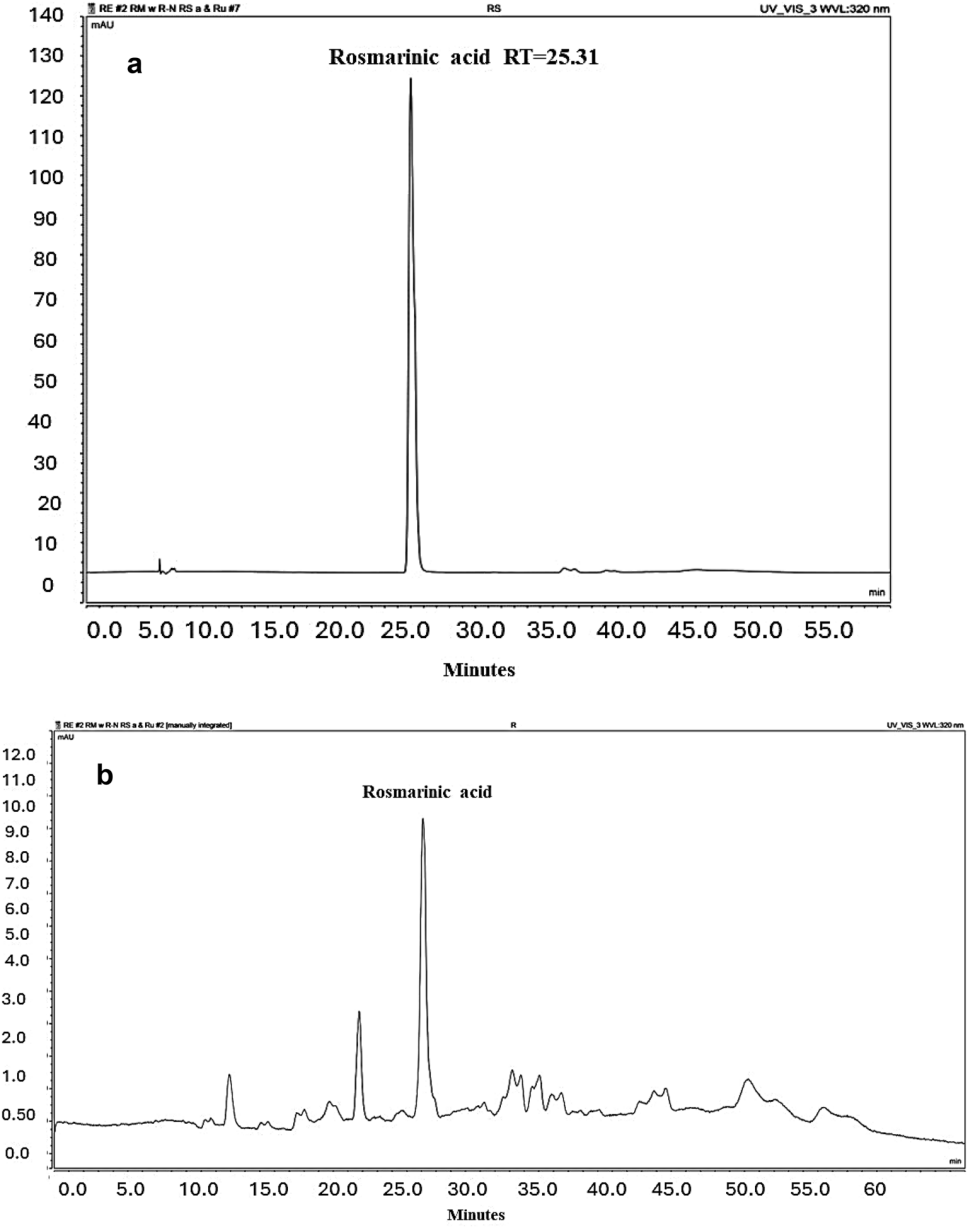
HPLC chromatograms; (**a**) Chromatogram of rosmarinic acid standard (Rt:25.31 min); (**b**) fingerprint chromatogram of rosemary herb ethanolic extract.

UPLC-PDA qualitative, a quantitative analysis of the ethanolic extract of neem and rosemary, correlates the presence of rutin and rosmarinic acid, respectively as a major identified peak. Compound identification in the chromatograms was achieved by matching their retention times and (UV) spectra to those of corresponding standards. Rutin peak was detected at 17 min (270 nm) while rosmarinic acid peak was observed at 25.31 min (320 nm) Figs. 1 and 2. By substituting in the standard calibration curves for rutin and rosmarinic acid; it is estimated that each 1 mg of rosemary extract contains 11.68058 µg of rosmarinic acid whereas 1 mg neem extract had 11.95076 µg of rutin.

### Anti-microbial activity

**Figure 3:**
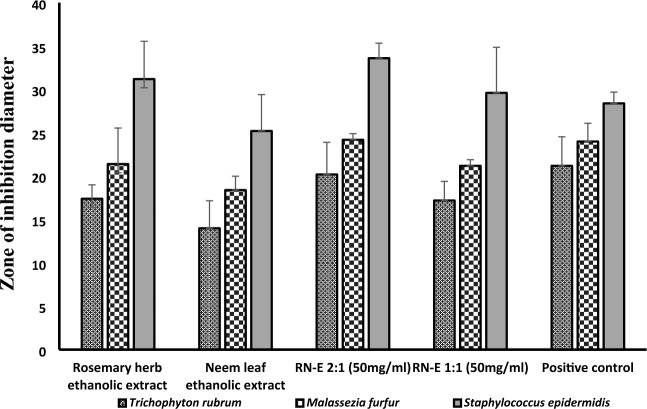
Antimicrobial effect of rosemary herb ethanolic extract, neem leaf ethanolic extract, (RN-E); rosemary -neem combined extract 2:1 and 1:1, and positive control; ketoconazole and gentamycin.

Figure 3 illustrates the comparative antifungal activity of various extracts against *Trichophyton rubrum* and *Malassezia furfur*. The combined extract, RN-E (2:1), exhibited the greatest zone of inhibition for both fungi (20.2 mm and 24.2 mm, respectively) compared to individual rosemary and neem extracts and the 1:1 combination of RN-E. The activity of RN-E (2:1) was comparable to the positive control, ketoconazole, for both fungal strains (21.2 and 24 mm respectively). Furthermore, RN-E (2:1) displayed the strongest antibacterial activity against *Staphylococcus epidermidis* (33.6 mm zone of inhibition), surpassing its 1:1 RN-E extract, and individual extracts. While gentamycin (positive anti-bacterial control) was 28.4 mm. It is noteworthy to mention that DMSO in which the extracts were dissolved had no efficacy against any of the tested strains when tested alone**.**

Based on the findings obtained, the RN-E (2:1) was the most effective against the tested organisms compared to RN-E (1:1), and the individual extracts. The MIC of RN-E (2:1) extract was determined using the broth microdilution method. The MIC of RN-E (2:1) extract was determined to be 6.25 mg/mL for *Trichophyton rubrum,* 12.5 mg/mL for *Malassezia furfur,* and 3.125 mg/mL for *Staphylococcus epidermidis.* Consequently, the combined extract RN-E (2:1) was selected for further evaluation**,** antioxidant assay, in-vitro anti-inflammatory assay, and development of potential pharmaceutical anti-dandruff and hair tonic formulations.

### Antioxidant activity

**Figure 4:**
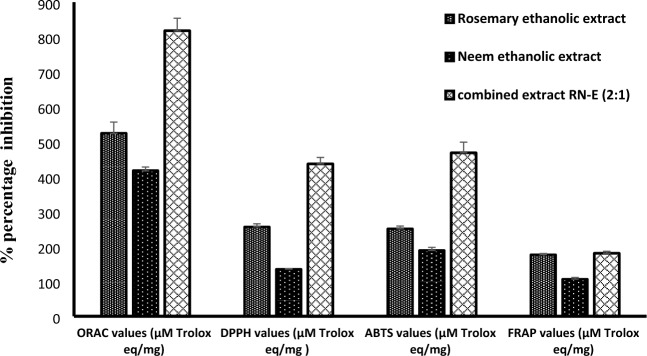
Comparative Antioxidant Activity of Rosemary Extract, Neem Extract, and their Combination RN-E (2:1) using ORAC, DPPH, ABTS, and FRAP Assays.

The antioxidant potential of the tested samples; rosemary extract, neem extract, and combined extract (2:1) was evaluated using different assays; DPPH free radical scavenging, oxygen radical absorbance capacity (ORAC), ferric reducing antioxidant power (FRAP), and ABTS free radical scavenging activity. As depicted in Fig. 4, the combined extract RN-E (2:1) exhibited the greatest antioxidant activity (818.596 ± 35.646, 436.71 ± 18.6, 468.436 ± 30.666, and 180.56 ± 2.593 μM Trolox equivalent/mg respectively)**,** potentially surpassing the activity of the individual extracts. This may potentially be explained by the high concentrations of flavonoids and phenolics present within the individual extracts.

### Anti-inflammatory activity

**Figure 5:**
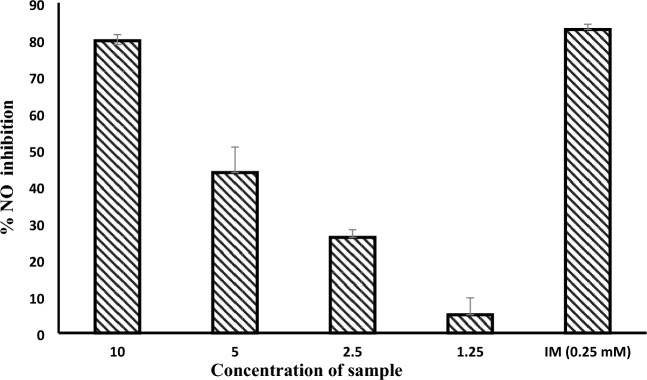
Concentration-Dependent Inhibition of LPS-Induced Nitric Oxide Release in RAW 264.7 Macrophages by Rosemary-Neem Extract (Compared to Indomethacin Control).

**Figure 6:**
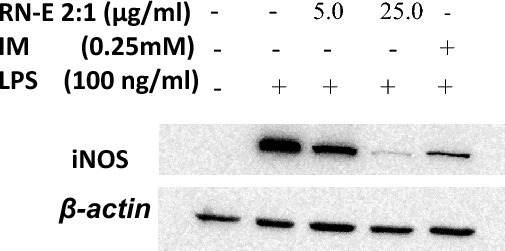
Impact of Rosemary-Neem Extract on iNOS and *β*-actin Protein Levels in LPS-treated RAW 264.7 Cells. RAW 264.7 cells were treated with 100 ng/mL of LPS only or with 5 and 25 μg/mL of rosemary -neem combined extract 2:1, or positive control; indomethacin (IM 25 mM) for 24 h.

Nitric oxide (NO) is released by murine macrophage RAW 264.7 cells as a response to Lipopolysaccharides (LPS) stimulation. Inducible nitric oxide synthase (iNOS) is a key mediator of immune activation which produces nitric oxide (NO). Hence, inhibitors of iNOS may be considered anti-inflammatory drug candidates^21,22^. The combined extract, RN-E (2:1), demonstrated significant inhibitory activity (89.16% ± 1.58) at a concentration of 10 µg/mL. Notably, this effect exceeded the activity observed with the positive control, indomethacin (81.20% ± 1.493), as detailed in Table 1. The combined extract (RN-E 2:1) showed concentration-dependent inhibition of LPS-induced nitric oxide (NO) release in RAW 264.7 macrophages (Fig. 5). The concentration of the sample that inhibits 50% of NO release was statistically derived using GraphPad Prism software, IC_50_ = 5.1 µg/mL. Furthermore, the mechanism of inhibition of NO secretion was done by finding the level of iNOS expression using Western blot (Fig. 6), Our findings indicate that the combined extract, RN-E (2:1), exhibited concentration-dependent inhibition of LPS-induced iNOS protein expression in macrophages.

**Table 1:** The percentage inhibition of NO.

%NO inhibition			
Conc. (µg/ml)	RN-E (2:1) (10 µg/ml)	RN-E (2:1) (100 µg/ml)	IM (0.25 mM)
	89.96	–	79.5
	90.16	–	81.8
	87.33	–	82.3
Mean	89.16	–	81.2
Std. deviation	1.58	–	1.493
Std. error of mean	0.912		0.8622

### Organoleptic properties evaluation

Organoleptic gel and leave-in hair tonic preparations of RN-E (2:1) were evaluated visually for homogeneity, color, shape, and odor. Overall, both formulae were light green, had pleasant odors, and showed homogeneity in appearance. The resultant herbal formulae were not transparent because the extract was dark green.

### PH measurements

The pH of the formulated herbal gel and leave-in hair tonic were 6.3 ± 0.021, and 6.5 ± 0.019, respectively. The standard pH of the scalp was around 4.5–6.5^23^, so these results indicate that the formulae are within a normal range of skin pH and will not produce any skin irritation or damaging the scalp.

### Viscosity evaluation

Viscosity, a measure of fluid resistance to flow, is inherently higher in gels, leading to their characteristic "thickness" and the term "gel preparation"^24^, this is obvious from the results of viscosity evaluation for the prepared formulae as the leave-in hair tonic preparation showed lower viscosity (5 cPs) than gel preparation (4000 cPs). This may indicate that the gel preparation will give better retention and deposition at the desired site.

### Spreadability of the herbal gel

The diameter of the predetermined circle was increased from 2 to 6 cm after applying weight indicating that the gel is easily spreadable. This supports the good quality of the gel.

### Ex-vivo skin deposition test

**Figure 7:**
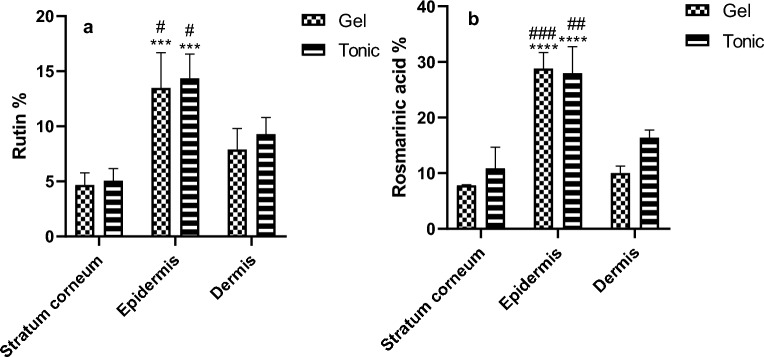
Deposited percent of rutin (**a**) and rosmarinic acid (**b**) from gel and tonic in each skin layer after 24 h. Results were compared using Two-way ANOVA followed by Sidak's post hoc test. ***p < 0.001 compared with respective rutin % deposited in the stratum corneum, #*p* < 0.05 compared with respective Rutin % deposited in the dermis. ****p < 0.0001 compared with respective rosmarinic acid % deposited in the stratum corneum, ##p < 0.01, ###*p* < 0.001 compared with respective rosmarinic acid % deposited in the dermis.

Figure 7 depicts the skin deposition profile of rosmarinic acid and rutin from each formulation across different skin layers (stratum corneum, epidermis, and dermis) over 24 h. Quantification of these ingredients was achieved using the previously validated UPLC-PDA method, ensuring no interference from residual skin components.

Formulating herbal (RN-E 2:1) extract in conventional gel and leave-in hair tonic, containing ethanol and propylene glycol, resulted in the augmented deposition of their active ingredients (rosmarinic acid and rutin) onto the stratum corneum. Furthermore, both formulations demonstrated significant penetration into the epidermis and dermis layers. The observed improvement in skin penetration for both preparations may be attributed to the presence of additional excipients, such as ethanol and propylene glycol, in the formulations. These excipients are widely recognized as penetration enhancers^25^. This assertion was supported by the absence of significant differences observed between the two dosage forms regarding the deposition of each active ingredient. It is worth saying that the significant deposition of both plant extracts (rosmarinic acid and rutin) is shown in the epidermis layer which is the place of *Malassezia* fungus.

### Hair growth activity test

The average length of the hair for each group was measured and revealed that, Gp-1(10.6 ± 0.11 mm), Gp-2 (19.4 ± 0.24 mm), Gp-3 (17.2 ± 0.06 mm), and Gp-4 (14.2 ± 0.33 mm). While the average thickness using the inverted microscope, for Gp-1, Gp-2, Gp-3, and Gp-4 were: 38.14 ± 0.89 µm, 68.39 ± 4.1 µm, 49.87 ± 1.87 µm, and 56.71 ± 2.03 µm, respectively. These results indicate that the length and thickness of the measured hairs about each formula were more than the untreated group and more than minoxidil 2% positive control.

## Discussion

Seborrheic dermatitis (SD) is a cutaneous disorder manifesting on areas with high concentrations of sebaceous glands, including the face, chest, and scalp. In adults, SD on the scalp is commonly known as dandruff (*pityriasis capitis*), while in infants, it’s termed cradle cap. Dandruff is characterized by the presence of parakeratotic cells, which can trigger an inflammatory response marked by the release of pro-inflammatory mediators. This results in the itching, redness, and flaking associated with the condition^26^. *Malassezia furfur* is strongly implicated as an etiological factor in dandruff due to its scalp lipase activity, which induces pro-inflammatory cytokine production and subsequent inflammation^27^. Conversely, *Trichophyton rubrum*, a fungus, causes Tinea capitis, a condition marked by itching, redness, and potential hair loss^28^. The exploration of natural products as potential avenues for promoting health and well-being is steadily increasing, largely driven by the recognition of their bioactive phenolic metabolites. These metabolites have been associated with antibacterial, antioxidant, and anti-inflammatory properties, potentially offering advantages over synthetic alternatives due to their reduced side effects. Consequently, research exploring innovative and cost-effective herbal formulations is becoming increasingly crucial. Notably, both neem leaves and rosemary herb extracts have been previously investigated and demonstrated promising antimicrobial activities, likely due to their richness in phenolic compounds^29–32^.

The present investigation sought to formulate two topical preparations, a gel, and a leave-in hair tonic, incorporating a standardized 2:1 ethanolic co-extract of rosemary herb and neem leaves (RN-E 2:1). This co-extract has demonstrated promising antimicrobial, antioxidant, and anti-inflammatory properties, suggesting its potential application in managing dandruff and hair loss. Mounting research has focused on the antimicrobial and anti-inflammatory properties of plant phenolics^33^. This study assessed the inhibitory effects of a combined rosemary and neem extract (RN-E) against *Malassezia furfur* and *Trichophyton rubrum*, microorganisms implicated in dandruff and hair loss. The findings revealed the superior efficacy of the (RN-E 2:1) extract compared to individual extracts of each plant. Additionally, our analysis demonstrated higher contents of phenolic and flavonoid in the combined extract compared to rosemary and neem alone. This result supports the reported correlation between antioxidant activity, and total phenolic and flavonoid contents in both neem and rosemary extracts, as demonstrated by Sinai et al. (2020)^34^. Notably, this study further suggests a synergistic enhancement of the antioxidant potential when the two extracts are combined comparable to Trolox as standard. HPLC–PDA standardization confirmed rosmarinic acid and rutin as the most prevalent constituents in the ethanolic extracts of rosemary and neem, respectively. Several studies stated that the main constituent of rosemary herb and neem ethanolic extract is rosmarinic acid, and rutin respectively^35^ These components potentially contribute to the observed antimicrobial activity^36^. Plant phenolics are widely recognized for their anti-inflammatory properties^37–40^. Rosemary and neem have established reputations for their anti-inflammatory effects, acting through diverse mechanisms. In this study, the RN-E 2:1 extract demonstrated concentration-dependent inhibition of LPS-induced macrophage iNOS protein production.

The incorporation of the herbal RN-E extract into conventional gel and leave-in hair tonic formulations resulted in the increased penetration of its active ingredients, rosmarinic acid, and rutin, into the stratum corneum. Furthermore, these components were observed to deposit deeply into the layer of the epidermis, the primary habitat of *Malassezia furfur* and *Trichophyton rubrum*. Notably, the assessed hair samples treated with the formulas exhibited greater length and thickness compared to the untreated control group. Additionally, these results surpassed the positive control, a 2% minoxidil solution**.** While ex-vivo studies revealed similar deposition concentrations of the active ingredients in both preparations across skin layers, the in-vivo application yielded superior results with the gel formulation. This may be attributed to the gel’s sustained release and adhesive properties, enabling prolonged contact with the scalp and potentially enhancing the penetration and activity of the components. This extended contact likely contributed to the observed improvement in hair growth and thickness compared to the leave-in tonic. These findings suggest that the herbal gel formulation might offer more pronounced effects on hair growth and thickness, despite the potential drawbacks of using a gel on the scalp. The observed improvements in hair parameters with the gel support its potential as a viable treatment option for hair loss and dandruff.

Our findings suggest that the inclusion of a standardized combination of rosemary herb and neem leaves within a pharmaceutical formulation warrants further exploration as a potential therapeutic strategy for managing dandruff and hair loss. However, comprehensive clinical trials are crucial for robust evaluation of the efficacy and safety of the developed formulation.

## Materials and methods

### Plant material

Neem leaves and Rosemary herbs were obtained from the Medicinal, Aromatic, and Poisonous Plants Experimental Station -Faculty of Pharmacy/Cairo University-October 2022). A flowering plant of both samples was identified and approved by Mrs. Therese Labib; Botanical Specialist and consultant at Orman and Qubba Botanical Gardens/Giza/Egypt. Voucher samples were submitted to the Department of Pharmacognosy, Faculty of Pharmacy, Cairo University, Giza, Egypt (voucher specimen RO-10-2021) for rosemary herb, and (voucher specimen AI-10-2021) for neem leaves.

### Chemicals

Rutin hydrate HPLC powder grade, rosmarinic acid, Folin-Ciocalteu reagent, 2,2-Diphenyl-1-picrylhydrazyl (DPPH), 2,2′ Azino-bis-(3-ethylbenzthiazoline-6-sulfonic acid; ABTS) in the crystallized diammonium salt form, 2,2′-Azobis (2-amidinopropane) dihydrochloride (AAPH), 6-hydroxy-2,5,7,8-tertamethylchroman-2-carboxylic acid (Trolox), fluorescein disodium salt, sodium, and TPTZ were purchased from (Sigma-Aldrich, St. Louis, MO, USA). Gallic acid and aluminum chloride were obtained from (Misr Company for Pharmaceutical Industry, Mataria, Cairo, Egypt). HPLC grade solvents, including methanol (MeOH) and acetonitrile, (Sigma-Aldrich, Steinheim, Germany) were employed, orthophosphoric acid (SD Fine Chem Ltd., Mumbai, India).

### Preparation of ethanolic extracts

Two kg of each plant was dried in the shade and ground to yield (1kg) powder. Each powder underwent separate extractions using repeated cold maceration in 90% ethanol for a 7-days duration until exhaustion was reached. The extracts were then filtered, combined, and evaporated to dryness via rotary evaporation under reduced pressure, maintaining a temperature below 50 °C. The final residue was about 61g (6.1% W/W) and 52.1g (5.2% W/W) from Rosemary and Neem ethanolic extract, respectively. Aliquots were used for phytochemical and biological studies.

### Evaluation of total phenolic and flavonoid contents

The total phenolic content of the extracts was quantified using a rapid microtiter plate Folin-Ciocalteu assay, as described by Attard et al.^41^. Additionally, the aluminum chloride assay was employed to screen the flavonoid content^42^. Gallic acid and rutin served as the standard references for phenolic acid and flavonoid, respectively. Briefly, sample preparation involved a 4 mg/mL solution in a 9:1 (v/v) methanol/water mixture. Calibration curves were established for gallic acid (25–1000 µg/mL) and rutin (5–160 µg/mL) through serial dilutions of their respective standards. Each curve was derived from the average of six replicates, achieving high correlation coefficients (R^2^) of 0.9994 for gallic acid and 0.9991 for rutin. (See Supplementary Fig. S1 online). Total phenolic content analysis involved the oxidation of samples with the Folin-Ciocalteu reagent at room temperature in sodium carbonate solution for 90 min. For flavonoid content, samples were reacted with aluminum chloride solution at room temperature. The resulting-colored products were measured using a microplate reader (FluoStar® Omega, BMG Lab tech, Germany) at 630 nm for gallic acid and 420 nm for rutin. Absorbance values were compared to standard curves generated using known concentrations of gallic acid and rutin. Results are expressed as µg gallic acid equivalents (GAE) per mg of dried sample for total phenolics and µg rutin equivalents per mg of dried sample for total flavonoid concentration. All data represent the mean ± SD of three independent replications.

### UPLC standardization

#### Stock preparation

Rosemary and neem ethanolic extract (10 mg) was accurately weighed and dissolved in methanol in 10 volumetric flasks to obtain a stock solution of 1 mg/mL and the same was done for the rutin and rosmarinic acid standards, yet 1 mL from the stock solution for each extract was transferred into 100 mL flask obtaining 100 µg/mL working stock solution. All samples were filtered through a 0.22 µm syringe filter and 10 µl was introduced to the column through autosampler (WPS-3000TRS, Thermo scientific USA).

#### Instrument

The analyses of rosemary herb and neem leaf extract were performed on Thermo Fisher UPLC Ultimate 3000–Complete UPLC (USA), equipped with a quaternary pump, degasser G1322A, and coupled with UV Detector (PDA -3000 RS, USA). Separation was achieved using a Hypersil Gold column (250 × 4.6 mm i.d., particle size, 5µ) at 25 °C. Agilent ChemStation software was used for data acquisition and processing.

#### Establishment of a standard calibration curve

Each standard compound (rutin and rosmarinic acid) was dissolved in methanol and used to establish calibration curves at concentration range (1–500 µg/mL). UV chromatographic profiles were analyzed, showing a rutin peak at 17 min (270 nm) while a rosmarinic acid peak was observed at 25.31 min (320 nm).

#### Method of the analysis

A mobile phase consisting of 0.1% (v/v) orthophosphoric acid in water as a solution (A) and Acetonitrile as a solution (B) was used at a flow rate of 1 ml/min for a 10 μL injection volume. Gradient elution ran for 55 min as follows: 0–3 min (15% B), 3–40 min (35% B), 40–50 min (15% B), followed by (100% B) for 5 min. The mobile phase in gradient flow was optimum to get uniform peak resolution of plant extract. The same gradient condition was used to determine rutin and rosmarinic acid calibration standards. Chromatographic profiles were UV detected at wavelength 270 nm for rutin in neem ethanolic extract and 320 nm for rosmarinic acid in rosemary ethanolic extract, each sample was injected in triplicates. The identification of rutin and rosamarinic acid peaks in the ethanolic extract of neem and rosemary was assessed depending on their retention time compared to that obtained for their corresponding standard as well as their UV spectra. Quantification of rutin and rosmarinic acid in the ethanolic extract of neem and rosemary ethanolic extract was done by comparing the peak areas of the samples with the calibration curves of corresponding standard solutions.

### Anti-microbial activity

#### Microorganisms

The fungal species utilised in this investigation included *Trichophyton rubrum* (RCMB 025002) and *Malassezia furfur* (RCMB 003), whereas the bacterial species encompassed *Staphylococcus epidermidis* RCMB 009. The microbes were acquired from the Regional Centre for Mycology and Biotechnology, Al-Azhar University (RCMB).

#### Agar well diffusion method

Susceptibility testing followed NCCL recommendations (National Committee Standards 1993). Using the agar well diffusion method, the antimicrobial activity of neem leaves, rosemary herb, and rosemary-neem extract combinations (RN-E 2:1) and (RN-E 1:1) was determined.

*Trichophyton rubrum* and *Malassezia furfur* cultures were spread over malt agar plates, while *Staphylococcus epidermidis* culture was spread over Mueller–Hinton agar plates. After spreading the culture, wells of 6mm diameter were cut in the agar surface with a sterile cork borer, and 100 µl of the tested extracts dissolved in dimethyl sulfoxide (DMSO) was added to these wells in a concentration of 50 mg/mL. ketoconazole (100 µg/ml) in DMSO was used as Positive control for fungi while, gentamycin (4 µg/ml) in DMSO for bacteria. DMSO alone was tested in wells. The plates were incubated for 48 h at 28 °C for fungi and 24 h at 37 °C for bacteria. To determine the antimicrobial activity, the diameter of the zone of inhibition was measured in mm.

#### Minimum inhibitory concentration assay (MIC)

The MIC of individual/combined rosemary-neem extracts that gave the highest zone of inhibition was evaluated by the broth microdilution method following CLSI recommendations (CLSI, 2008). Serial dilution of extract dissolved in DMSO was prepared in a range from (25–0.125 mg/mL). Following incubation (48 h at 28 °C for fungi and 24 h at 37 °C for bacteria), the minimum inhibitory concentration (MIC) of the extract was determined. The MIC was established as the lowest extract concentration exhibiting no visible microbial growth in the wells^43^.

### Assessment of antioxidant activity

#### DPPH free radical scavenging assay

The DPPH free radical scavenging assay was conducted on the two individual extracts and RN-E (2:1) combined extract according to the procedure previously stated by Chen et al.^44^. Briefly, the individual extracts and (RN-E 2:1) extract were prepared to obtain a final concentration of 0.05 mg/mL in methanol, a standard stock solution of Trolox at a concentration of (100 µM) in methanol was serially diluted to six concentrations; (5–40 µM). Freshly prepared DPPH reagent 100 µL (0.1% in methanol) was added to 100 µL of each sample in a 96-well plate (n = 6), and the reaction was incubated at room temp for 30 min in the dark. Following incubation, the reduction in DPPH radical activity was quantified by measuring the decrease in absorbance at 540 nm using a microplate reader^45^. The results were expressed as micromolar (μM) Trolox equivalents per milligram (mg) of the sample, calculated using the linear regression equation derived from the calibration curve (see Supplementary Fig. S2).

#### Oxygen radical absorbance capacity (ORAC) assay

The oxygen radical absorbance capacity (ORAC) assay, adapted with slight modifications from the method described by Liang et al.^46^, was employed to assess the antioxidant activity of the samples. Briefly, the assay was conducted in black 96-well plates (Corning Scientific, Corning, NY). Each well contained 10 µL of prepared samples at a concentration of 200 µg/mL in methanol, or 10 µL of Trolox standard solution (final concentration 2 mM, with a range of 50–800 µM). Additionally, 30 µL of fluorescein (final concentration 100 nM) was added to each well. The plates were then incubated at 37 °C for 10 min. Fluorescence measurements were performed for background assessment using three cycles (90 s each) at an excitation wavelength of 485 nm and an emission wavelength of 520 nm. Subsequently, 70 µL of freshly prepared 2,2′-Azobis(2-amidinopropane) dihydrochloride (AAPH) solution (240 mM) was promptly added to each well. Fluorescence measurement was then continued for 60 min at the same excitation and emission wavelengths, employing 40 cycles (90 s each).

ORAC values, expressed as µM Trolox equivalents (TE) per milligram (mg) of the sample, were calculated using the linear regression equation derived from the calibration curve (See Supplementary Fig. S3 online), depicting the linear dose-inhibition curve of Trolox). Data are presented as mean ± SD, based on triplicate measurements.

#### Determination of ferric reducing antioxidant power (FRAP) assay

This method leverages the ability of the samples, at low pH, to reduce ferric (Fe^3+^) ions to ferrous (Fe^2+^) ions. In the presence of TPTZ (2,4,6-Tris(2-pyridyl)-s-triazine) [Sigma Aldrich, USA], the formed Fe^3+^-TPTZ complex is reduced to the Fe^2+^-TPTZ complex, generating an intense blue color with a maximum absorbance at 593 nm. The assay is adapted from the method of Benzie and Strain^47^, with slight modifications for microplate compatibility. Briefly, samples were prepared at 25 mg/mL in methanol, while the standard Trolox solution (2 mM in methanol) was serially diluted within the range of 1500–50 µM. Each well of a 96-well plate was filled with 190 µL of freshly prepared TPTZ reagent (prepared by mixing 300 mM acetate buffer (pH 3.6), 10 mM TPTZ in 40 mM HCl, and 20 mM FeCl_3_ in a 10:1:1 v/v/v ratio) followed by 10 µL of the sample. Triplicates were prepared and measured for each sample. The reaction mixture was incubated in the dark for 30 min at room temperature. After incubation, the absorbance of the resulting blue color was measured at 593 nm using a microplate reader. The ferric-reducing ability of the samples was expressed as µM Trolox equivalents (TE) per mg of sample, calculated using the linear regression equation derived from the calibration curve (see Supplementary Fig. S4 online). Data are presented as mean ± SD.

#### Free radical scavenging activity (ABTS) assay

The assay was carried out adopting the method of Arnao et al.^48^, with minor modifications. Briefly, an ABTS stock solution was prepared by dissolving ABTS (Sigma Aldrich, USA) in DW and adjusting the volume to 25 mL. Subsequently, 1 mL of this solution was mixed with 17 µL of 140 mM potassium persulfate and incubated in the dark for 24 h. The reaction mixture was then diluted with methanol to a final volume of 50 mL to obtain the working ABTS solution. In a 96-well plate, 190 µL of the freshly prepared ABTS solution was mixed with 10 µL of the samples (prepared at 0.5 mg/mL in ethanol) or Trolox standard solutions (2 mM, diluted in methanol to a range of 600–50 µM) (n = 6) (see Supplementary Fig. S5 online). The reaction mixture was incubated in the dark for 30 min at room temperature. After incubation, the decrease in absorbance of the ABTS radical cation was measured at 734 nm using a microplate reader.

The percentage inhibition of ABTS radical cation by each extract was calculated using the following equation and expressed as mean ± SD:

$$ \% {\text{ Inhibition}}\,\left( {I\% } \right)\, = \,[\left( {{\text{Average}}\,{\text{absorbance}}\,{\text{of}}\,{\text{blank}}\, - \,Average\,absorbance\,of\,average\,absorbance\,of\,bank \, } \right) \times 100 $$

### Anti-inflammatory in vitro activity in Murine macrophage cell line RAW 264.7 cells activated with lipopolysaccharide

#### Cell culture and media

Murine macrophage RAW264.7 cells (ATCC®) were cultured in a humidified incubator containing 5% CO_2_. The culture medium consisted of complete DMEM (Corning, USA) supplemented with 10% fetal bovine serum, 100 U/mL penicillin, 100 µg/mL streptomycin sulfate, and 2 mM L-glutamine. For subculture and treatments, cells were detached from the flasks using sterile cell scrapers.

#### Inhibition of LPS-induced NO release

RAW 264.7 cell stock (0.5 × 106 cells/mL) was inoculated into 96-well microwell plates and incubated overnight. On the next day, cells received either DMSO, 0.1% v/v considered as the negative control group [LPS−], lipopolysaccharide (Sigma-Aldrich, from Escherichia coli serotype O111: B4) at a concentration of (100 ng/mL) assigned as inflammation group [LPS +], LPS in the presence of the combined extract (RN-E 2:1) at screening concentrations of (10 and 100 µg/mL) dissolved in DMSO and diluted into culture media (final concentration of DMSO = 0.1%, by volume) that was considered as sample group, or indomethacin (IM, 0.25 mM) for anti-inflammatory positive control group. Using the Griess reaction^49^ test for the determination of nitric oxide levels in quadrants of culture supernatants after 24 h of exposure in all groups (n = 3).

#### NO assay

Briefly, equal volumes (100 µL) of culture supernatants and Griess reagent, prepared as described previously^49^ containing (1% sulfanilamide, 0.1% naphthyl ethylene diamine dihydrochloride, and 2.5% phosphoric acid), were mixed and incubated at room temperature for 10 min. The resulting colored diazonium salt was measured for absorbance at 540 nm using a microplate reader (Tecan Sunrise™, Austria). Nitric oxide (NO) inhibition percentage (%) of the tested extract (RN-E 2:1) was calculated relative to the LPS-stimulated control group ([LPS +]) and normalized to viable cell number, as determined by the Alamar Blue™ reduction assay^50^.

#### Rosemary-Neem combined extract concentration-dependent iNOS

RAW 264.7 cells stock (0.75 × 10^6^ cells/mL) were seeded into 6-well plates and incubated overnight. Following the protocol described above, triplicate wells in the sample groups received increasing concentrations (1.25, 2.5, 5, and 10 µg/mL) of the dissolved sample (RN-E 2:1) in DMSO, diluted into culture medium containing LPS (final DMSO concentration 0.1% v/v). After 24 h of incubation, the NO content in all wells was determined using the Griess assay, as previously described^49^ The concentration inhibiting 50% of LPS-induced NO release (IC_50_) was statistically calculated using GraphPad Prism software, employing a non-linear regression model curve fit.

#### iNOS by western blot analysis

Overnight culture of RAW264.7 (6-well plates, initially seeded as 1.5 × 106 cells/well) was treated with (RN-E 2:1) combined extract (5 and 25 µg/mL), or indomethacin (IM positive control, 250 μM). After 24 h of incubation, Raw 264.7 cells were washed using cold PBS (phosphate buffered saline) and scraped from each well in cold lysis buffer [RIPA buffer containing protease inhibitor cocktail, 100 µl/well]. Cell lysates were incubated on ice for 15 min with an occasional vortex every 5 min. Cell lysates were centrifuged at 10,000×*g* for 10 min (4 °C). Total protein was determined in the supernatants using a BCA kit (Thermofisher Scientific, USA). Samples (20 µg total proteins) were resolved using polyacrylamide gel electrophoresis on a 10% acrylamide/bis-acrylamide gel using the OmiPAGE TETRAD system (Cleaver Scientific, UK) at 150 V for 1.5 h. A nitrocellulose membrane was electro-transferred with resolved proteins at 100 V for 70 min. Membranes were blocked using skim milk (5% w/v in TS buffer saline, Tween 20, TBST) for 1 h at room temperature. Membranes were probed overnight (4 °C) with a rabbit iNOS antibody (Biomatik, Canada) and *β*-actin (Thermofisher Scientific, USA) under gentle shaking. Probed membranes were washed with TBST for 3 × 5 min under shaking. Membranes were then incubated for 1 h at room temperature with matched horseradish peroxidase-conjugated secondary antibodies under gentle shaking. Using an enzyme chemiluminescence reagent (Clarity, Bio-Rad, USA), protein bands were observed following a TBST washing period of 3 × 5 min.

### Preparation of the pharmaceutical formulae

#### Materials

Carbopol 934, methylparaben, and propylparaben of analytical grade and sodium hydroxide pellets were purchased from Adwic Co. (Cairo, Egypt). Propylene glycol, Jasmine oil, and absolute ethanol were purchased from Agitech Co. (Cairo, Egypt).

#### Formulation of herbal gel, and leave-in hair tonic

Two types of formulae were made using plant extract (RN-E 2:1) as shown in (Table 2). The herbal gel was formulated by adding the extract to propylene glycol till a completely homogenous mixture was obtained. On a magnetic stirrer, 1% of carbopol 934 was sprinkled on the surface of the previous mixture and left till swelling of the polymer. The obtained gel was then neutralized using a few drops of triethanolamine till the desired consistency was achieved.

**Table 2:** Plant extract formulation composition (gel and hair tonic).

Ingredients	Gel	Leave-in hair tonic
	(% w/w)	
Carbopol 934	1	–
Propylene glycol	5	5
Ethanol absolute	5	5
Jasmine oil	–	5
Methyl paraben	0.2	0.2
Propyl paraben	0.02	0.02
Distilled water (up to)	100	100

1% of (RN-E-a) has been added to each preparation.

As for the leave-in hair tonic, RN-E was dissolved in jasmine oil (Table 2). The solution was then mixed with propylene glycol. After that, it was added portion-wise to distilled water and stirred on a magnetic stirrer to obtain a homogenous one-phase leave-in hair tonic. Finally, a few mL of absolute ethyl alcohol was added to the hair tonic to enhance the homogeneity of the tonic.

### Evaluation of herbal gel, and leave-in hair tonic

To evaluate the formulated herbal gel and leave-in hair tonic, several quality tests were conducted including organoleptic properties, viscosity, and pH determination. Furthermore, Ex-vivo skin deposition testing for hair tonic and herbal gel was done. While the spreadability testing was done for herbal gel formula only.

#### The organoleptic examination

Both plant extract formulations were subjected to an organoleptic evaluation, which included assessing their color, odor, and homogeneity through visual observation.

#### PH measurement

The pH of the RN-E (2:1) herbal gel and leave-in hair tonic were determined using a pH meter (Jenway, 3510 pH meter) at room temperature.

#### Viscosity determination

The viscosity of the formulated gel and leave-in hair tonic was determined using Brookfield Viscometer (Ametek, DV-1 viscometer) at room temperature.

#### Spreadability evaluation of herbal gel

The spreadability of the formulated gel was determined by spreading 0.5 gm of the gel on a circle having a 2 cm diameter predetermined on a glass plate and another glass plate was placed on top. 50 g weight was applied on the upper glass plate and allowed to rest for 5 min and then the spreadability of the gel was determined by measuring the new diameter of the circle.

### Ex-vivo skin deposition testing of herbal gel and leave-in hair tonic

#### Animals

Female Swiss albino mice (20–25 g)/Wistar rats weighing (150–200 g) were obtained from the animal house (Faculty of Pharmacy/The British University in Egypt). Mice were housed in a temperature-controlled environment (25 °C) with a light–dark cycle of 12 h every day and were allowed free access to standard laboratory food (standard diet and tap water ad libitum). This study is approved by the ethics committees of the Committee of Animal Care and Use of NRC. All procedures and experiments were performed according to the approved protocol, and animals were treated in accordance with ARRIVE guidelines (https://arriveguidelines.org). The British University in Egypt's Faculty of Pharmacy and Ethical Committee accepted the experiment under reference number EX-2207.

#### Removal of skin

Two mice were used for each formula, the skin was carefully removed after euthanizing the female mice (humane killing) using a CO_2_ chamber (See Supplementary Fig. S6 online). The skin was removed from both dorsal and ventral sides, and subcutaneous tissues and adhering fats were removed by rubbing with a cotton swab. Before beginning the experiment, the excised full-thickness skin samples were equilibrated by soaking them in phosphate buffer saline (PBS) solution with a pH of 7.4 at 4–8 °C for approximately one hour. Each skin was kept separately in the refrigerator for further ex-vivo skin deposition study. The dead bodies were discarded by incineration.

#### Ex-vivo skin deposition test

Ex-vivo skin testing was carried out using static vertical Franz diffusion cells with a diffusion area of 1.77 cm^2^. The receptor compartment of the Franz cells was filled with pH 7.4 phosphate buffer and maintained at 37.5 °C water bath. The dorsal skin of mice was used as the membrane. The skin was placed with the stratum corneum facing the donor compartment. After equilibration for 30 min. A 0.5 g of gel or 500 uL of hair tonic containing the same concentration of plant extract was applied to the stratum corneum and the Franz cells were left for 24 h on a magnetic stirrer at 100 rpm. At the end of deposition testing, the dorsal skin of the mice was removed from the Franz cells and gently wiped to remove formulation residue. The skin was then stripped 20 times using an adhesive tape to remove the stratum corneum layer. Afterward, the epidermis and dermis were separated using a pair of forceps, and each layer was cut into small pieces. Each skin layer was then soaked using 20 mL of methanol and the collected samples were subjected to analysis using validated UPLC-PDA (as previously mentioned), subsequently, the samples were filtrated through 0.22 µm filter membrane to determine the drug concentration hence deposition in each layer. The experiment was conducted in triplicate and the results were calculated as mean ± SD.

### Statistical analysis

GraphPad Prism software (GraphPad Software Tools, Inc., La Jolla, CA, USA) was used in statistical analyses. Results were compared using Two-way ANOVA followed by Sidak's post hoc test with a statistically significant difference at *p* < 0.05.

### Hair growth activity test on rats

Twenty rats were randomly divided into four treatment groups, each group consisting of 5 rats. The first group (Gp-1) was left untreated (control group), The second group (Gp-2) was treated with (RN-E 2:1) hair gel 1%, the third group (Gp-3) was treated with (RN-E 2:1) leave-in hair tonic 1%, and the fourth group (Gp-4) (positive control group), that applied minoxidil spray 2% as a hair tonic. Following hair removal by shaving 5 × 5 cm^2^ of the dorsal skin of the rats, the residual hairs were removed using depilatory cream. In each testing area, a square measuring 2 × 2 cm^2^ was outlined. Then, the rats were left for 24 h. After that, samples were applied topically to the test area once daily for 30 consecutive days at a volume of 2 mL (see Supplementary Fig. S8 online). The rats’ hair was evaluated by measuring the average hair’s length using a caliper and inverted microscope (in μm) to measure the thickness.

## Conclusion

This study presents the development of gel and leave-in anti-dandruff and hair tonic formulations utilizing a standardized, combined ethanolic extract of rosemary herb and neem leaves. The formulations capitalize on the extract's demonstrated antifungal and antibacterial properties, addressing scalp disorders. The prepared formulae exhibited significant antioxidant and anti-inflammatory activities, likely due to their high phenolic and flavonoid content. Both qualitative (quality control tests) and quantitative (skin deposition) evaluations yielded positive results, further supporting the formulations’ potential. In-vivo studies demonstrated promising results for the hair tonic effect. However, comprehensive in-vitro antimicrobial testing and clinical trials are strongly recommended for robust evaluation of the formulae's efficacy and safety.

### Supplementary Information

Supplementary Figures.

## Data Availability

All data generated or analyzed during this study are included in this published article [and its supplementary information file].
