# Supplementary material for: Rosemary and neem: an insight into their combined anti-dandruff and anti-hair loss efficacy
Source: Sci Rep. 2024 Apr 2;14:7780. doi: 10.1038/s41598-024-57838-w (PMC10987638; doi:10.1038/s41598-024-57838-w)
Supplement: Supplementary file 1 — Supplementary Figures. [file 41598_2024_57838_MOESM1_ESM.pdf]

Rosemary and Neem: An insight into their combined anti-dandruffand and anti-hair loss efficacy

Mona M. Hashem *[1], Dalia Attia [2] , Yomna A Hashem [3], Moataz S Hendy [4,5], Safa AbdelBasset [6], Farah Adel [2], Maha M Salama [1,6]

1 Department of Pharmacognosy, Faculty of Pharmacy, Cairo University, Kasr El-Aini Street, Cairo 11562, Egypt

2 Department of Pharmaceutics and Pharmaceutical Technology, Faculty of Pharmacy, The British University in Egypt, Suez Desert Road, El Sherouk City, Cairo, 11837, Egypt

3 Department of Microbiology, Faculty of Pharmacy, The British University in Egypt, El Sherouk City, Suez Desert Road, Cairo 11837, Egypt

4 Department of Pharmaceutical Chemistry, The British University in Egypt, El Sherouk City, Suez Desert Road, Cairo 11837, Egypt

5 Health Research Centre of Excellence, Drug Research and Development, The British University in Egypt, El Sherouk City, Suez Desert Road, Cairo 11837, Egypt

6 Department of Pharmacognosy, Faculty of Pharmacy, The British University in Egypt, El Sherouk City, Suez Desert Road, Cairo 11837, Egypt

* mona.hashem@pharma.cu.edu.eg

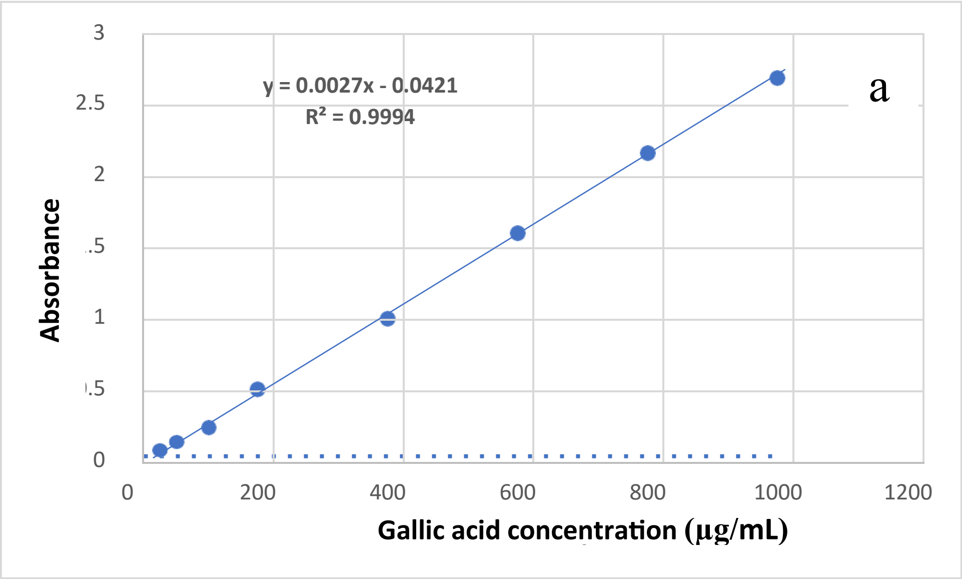

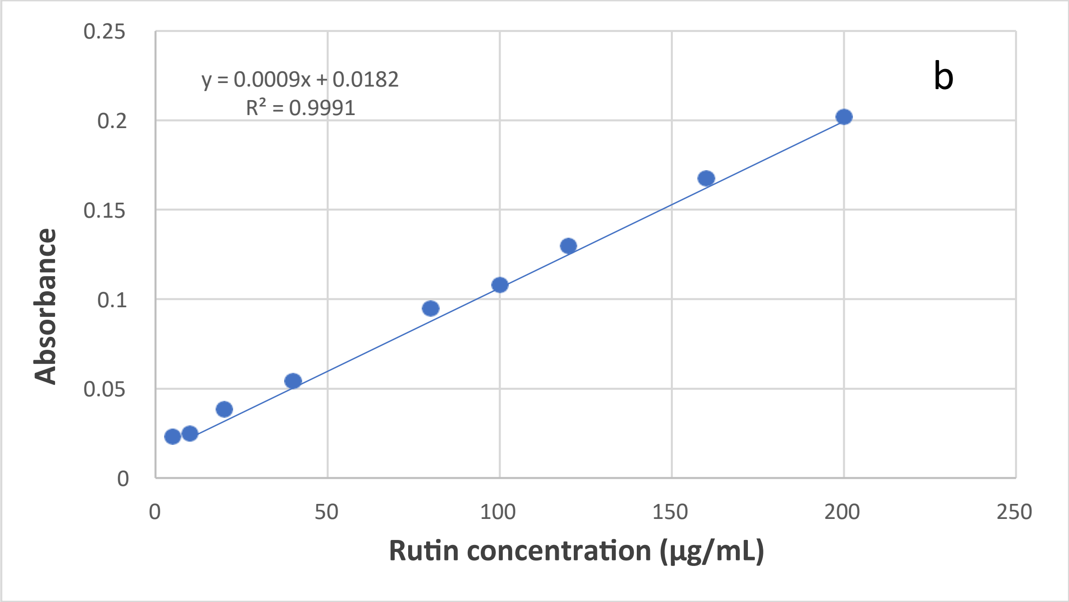

Figure S1. Standard Curves of (a) Gallic Acid and (b) Rutin (Mean of 6 Replicates)

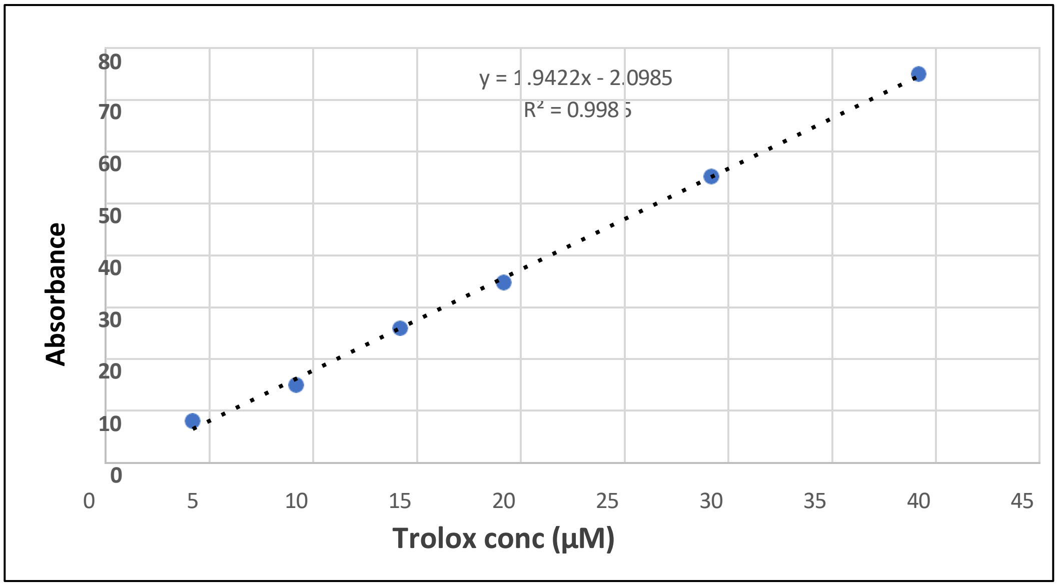

Figure S2. Standard Curve of Trolox for DPPH Assay (Mean of 6 Replicates).

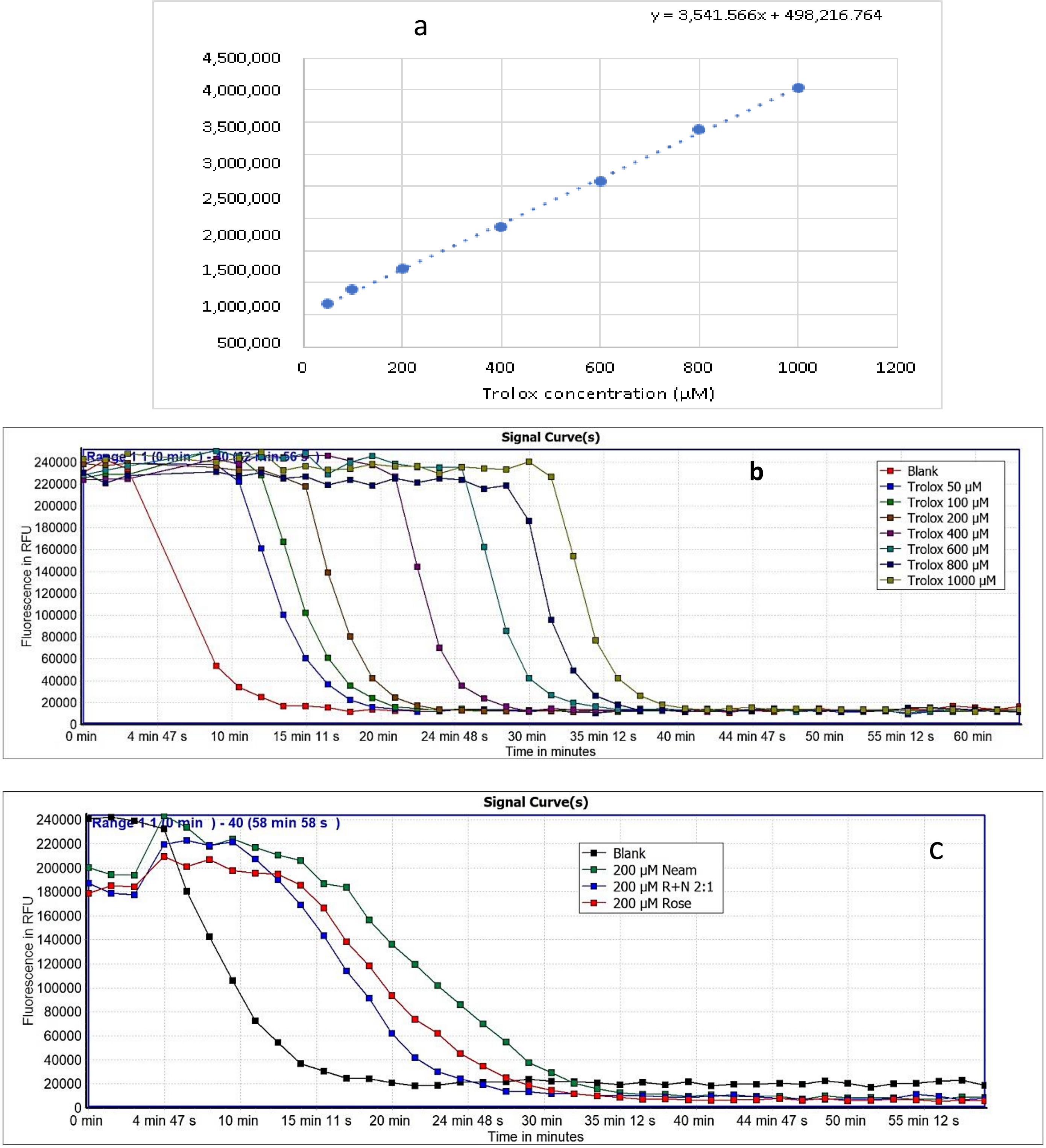

Figure S3. a) Standard Curve of Trolox for ORAC Assay (Mean of 6 Replicates), b) Signal curves of different Trolox concentrations and blank, indicating the decay of fluorescein with different concentrations of Trolox, c) Signal curve of Samples and blank indicating the decay of fluorescein upon applying the samples

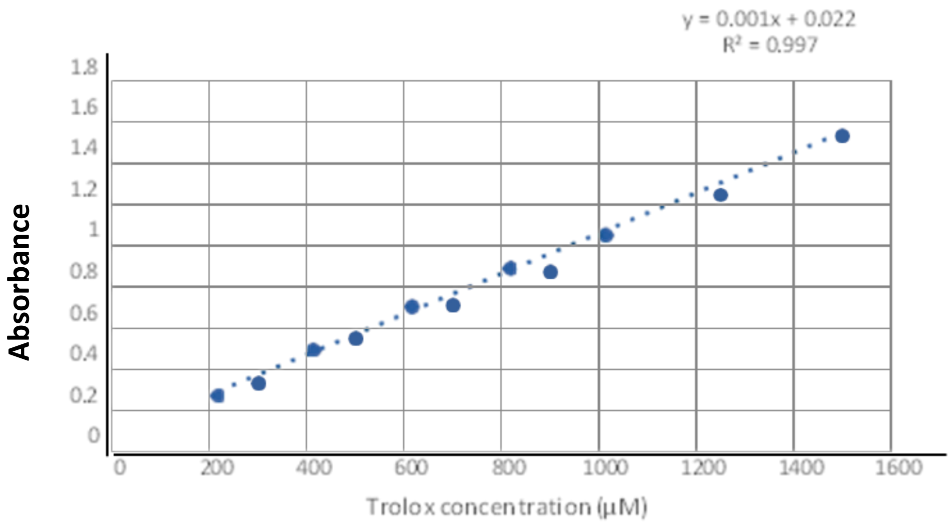

Figure S4. Standard Curve of Trolox for FRAP Assay (Mean of 6 Replicates).

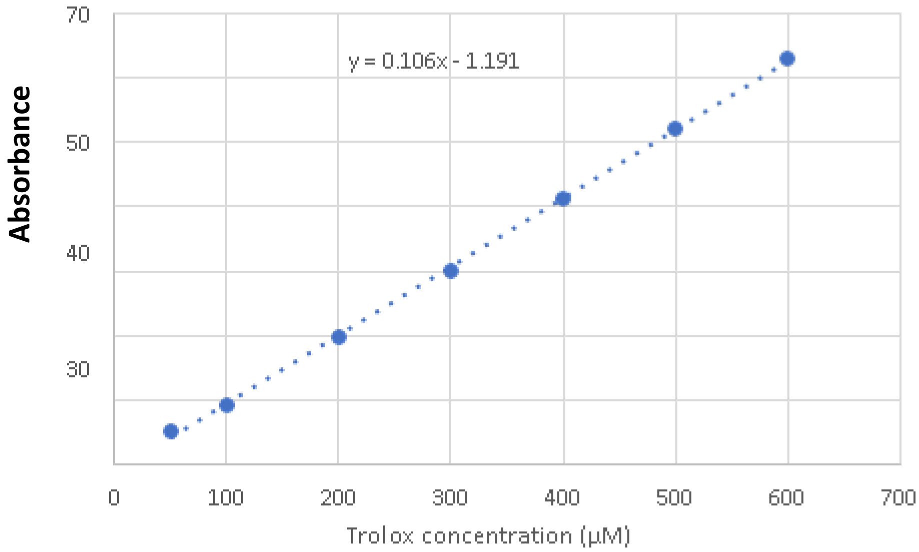

Figure S5. Standard Curve of Trolox for ABTS Assay (Mean of 6 Replicates)

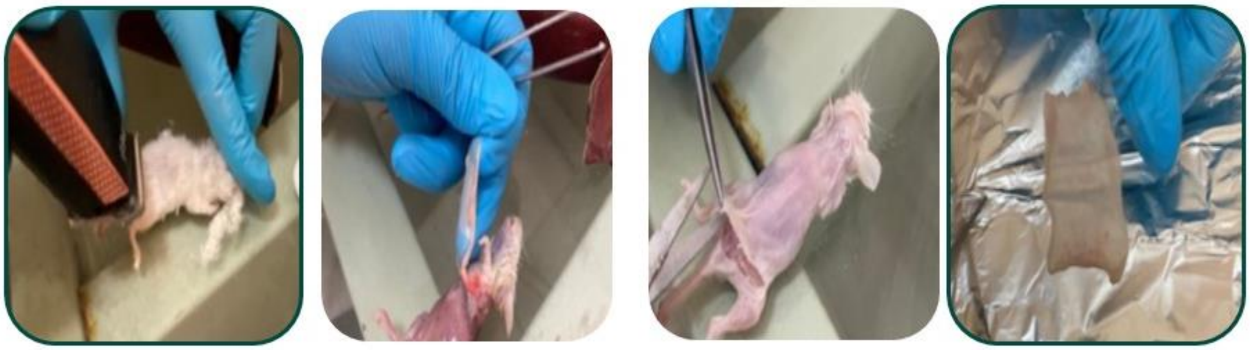

Figure S6. Removal of the skin for *ex vivo* skin deposition test

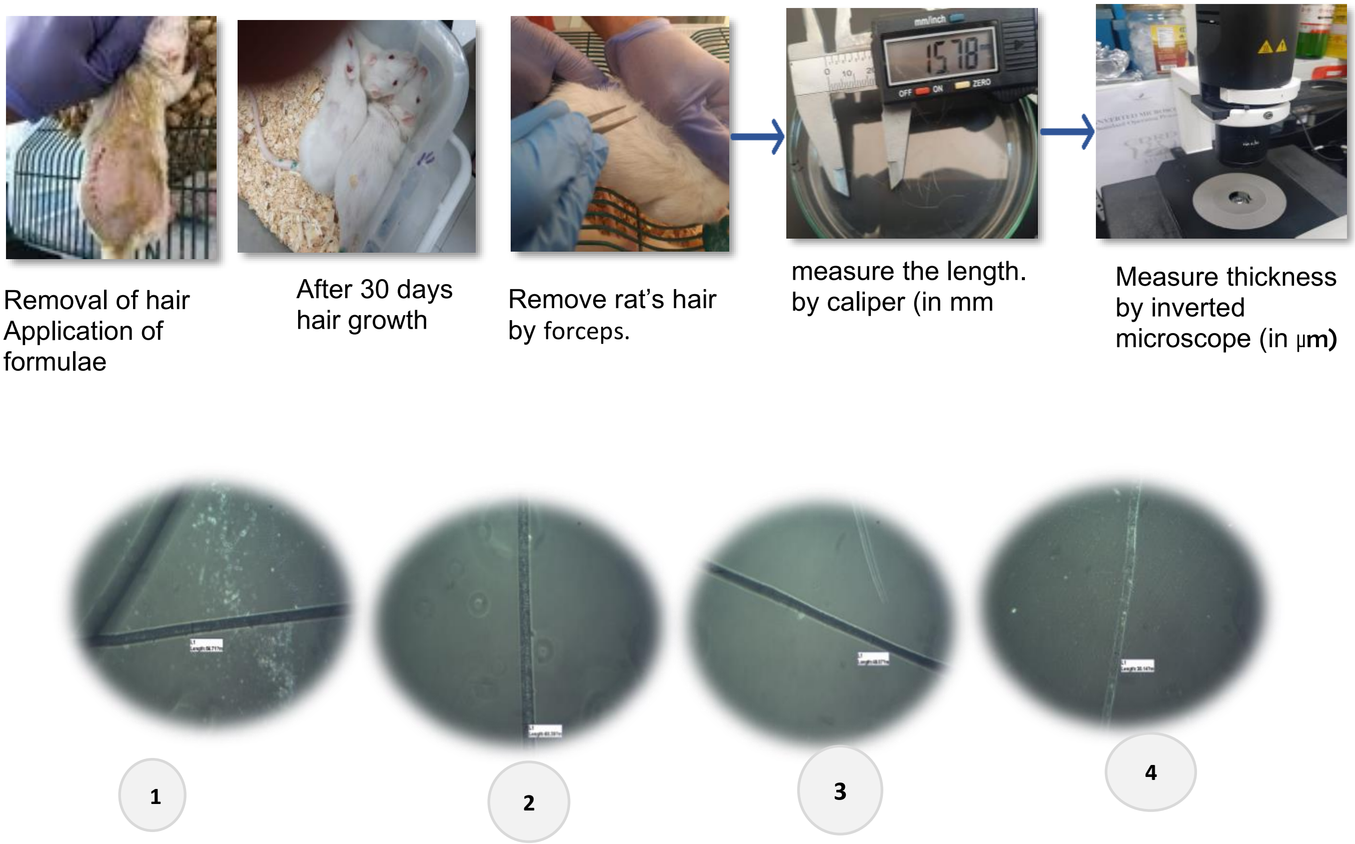

****The average of 4 measurements*

Figure S7. Hair growth test; Measurement of hair length by the caliper and hair thickness by inverted microscope .

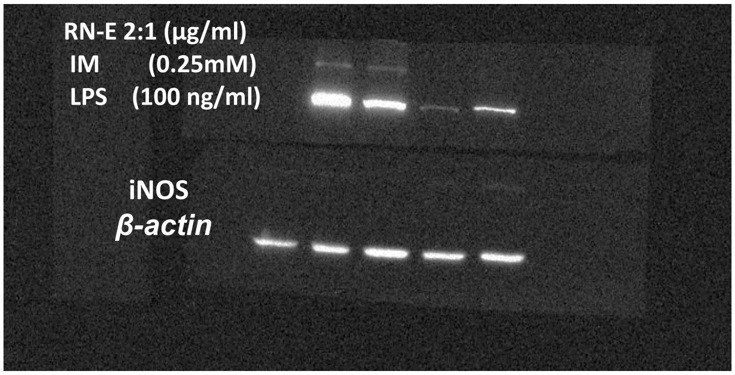

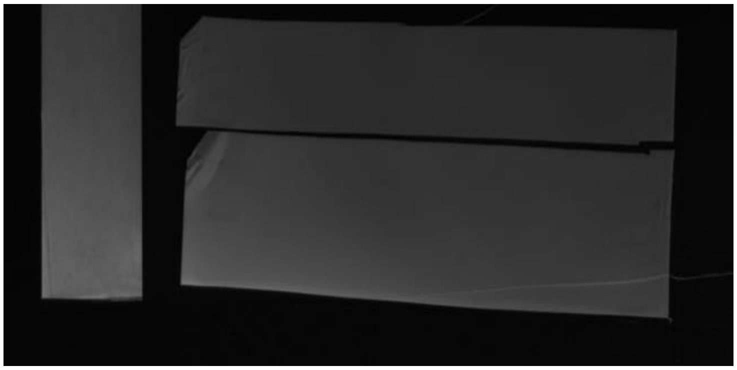

Spliced Western blot image of LPS-induced iNOS protein expression.
